# Transcriptomic meta-analysis reveals ERRα-mediated oxidative phosphorylation is downregulated in Fuchs’ endothelial corneal dystrophy

**DOI:** 10.1371/journal.pone.0295542

**Published:** 2023-12-14

**Authors:** Xunzhi Zhang, Ashwani Kumar, Adwait A. Sathe, V. Vinod Mootha, Chao Xing

**Affiliations:** 1 Eugene McDermott Center for Human Growth and Development, University of Texas Southwestern Medical Center, Dallas, Texas, United States of America; 2 Department of Ophthalmology, University of Texas Southwestern Medical Center, Dallas, Texas, United States of America; 3 Department of Bioinformatics, University of Texas Southwestern Medical Center, Dallas, Texas, United States of America; 4 O’Donnell School of Public Health, University of Texas Southwestern Medical Center, Dallas, Texas, United States of America; L.V. Prasad Eye Institute, INDIA

## Abstract

**Background:**

Late-onset Fuchs’ endothelial corneal dystrophy (FECD) is a degenerative disease of cornea and the leading indication for corneal transplantation. Genetically, FECD patients can be categorized as with (RE+) or without (RE-) the CTG trinucleotide repeat expansion in the transcription factor 4 gene. The molecular mechanisms underlying FECD remain unclear, though there are plausible pathogenic models proposed for RE+ FECD.

**Method:**

In this study, we performed a meta-analysis on RNA sequencing datasets of FECD corneal endothelium including 3 RE+ datasets and 2 RE- datasets, aiming to compare the transcriptomic profiles of RE+ and RE- FECD. Gene differential expression analysis, co-expression networks analysis, and pathway analysis were conducted.

**Results:**

There was a striking similarity between RE+ and RE- transcriptomes. There were 1,184 genes significantly upregulated and 1,018 genes significantly downregulated in both RE+ and RE- cases. Pathway analysis identified multiple biological processes significantly enriched in both—mitochondrial functions, energy-related processes, ER-nucleus signaling pathway, demethylation, and RNA splicing were negatively enriched, whereas small GTPase mediated signaling, actin-filament processes, extracellular matrix organization, stem cell differentiation, and neutrophil mediated immunity were positively enriched. The translational initiation process was downregulated in the RE+ transcriptomes. Gene co-expression analysis identified modules with relatively distinct biological processes enriched including downregulation of mitochondrial respiratory chain complex assembly. The majority of oxidative phosphorylation (OXPHOS) subunit genes, as well as their upstream regulator gene estrogen-related receptor alpha (*ESRRA*), encoding ERRα, were downregulated in both RE+ and RE- cases, and the expression level of *ESRRA* was correlated with that of OXPHOS subunit genes.

**Conclusion:**

Meta-analysis increased the power of detecting differentially expressed genes. Integrating differential expression analysis with co-expression analysis helped understand the underlying molecular mechanisms. FECD RE+ and RE- transcriptomic profiles are much alike with the hallmark of downregulation of genes in pathways related to ERRα-mediated OXPHOS.

## Introduction

Fuchs’ endothelial corneal dystrophy (FECD) is an age-related degenerative disease of cornea that can lead to significant vision loss. It is characterized by the progressive loss of the normal morphology and cell density of the corneal endothelium accompanied by diffuse thickening of Descemet’s membrane with focal excrescences called guttae [1]. FECD can be categorized into early-onset and late-onset subtypes according to the time point it becomes clinically evident. The rare early-onset FECD usually happens in the first decade of life, whereas the more prevalent late-onset type occurs in the fourth to fifth decade [2]. The prevalence of the late-onset FECD was estimated to be >4% of the population over the age of 40 and increased with age [3, 4]. It is the leading indication for corneal transplantation in the United States and worldwide [5, 6].

FECD is a complex disease hypothesized to involving both genetic and environmental factors and interaction between them [7]. Variants in 10 genes/loci (*AGBL1*, *COL8A2*, *LOXHD1*, *SLC4A11*, *ZEB1*, *DMPK*, *TCF4*, *KANK4*, *LAMC1* and *ATP1B1*/*LINC00970*) have been shown to be either causal or highly associated with the disease [8–15]. The extended CTG trinucleotide repeat expansion (CTG18.1) in *TCF4* was determined to be causal for the disease [16] and estimated to explain two-thirds of cases [17] with several plausible pathogenic mechanisms proposed [18–25]. It was shown the expanded CUG RNA binds and sequesters critical RNA splicing factors such as MBNL1 and MBNL2, leading to broad dysregulation of splicing of MBNL-sensitive exons [18, 23, 24]. However, except for *DMPK* the remaining genes do not harbor repeat expansions nor have obvious interconnections or shared common pathways. Thus, the mechanisms underlying FECD remain to be fully elucidated. As a starting point, it would be instructive to identify the similarities and differences of molecular changes between FECD patients with (RE+) and without (RE-) CTG18.1 expansions.

Transcriptomic profiling by RNA sequencing (RNA-seq) enables an overview of the molecular processes involved in FECD. There have been a few RNA-seq studies of corneal endothelium in late-onset FECD patients and controls published [18, 22, 23, 26]. As results from a single RNA-seq experiment may be to subject to bias and insufficiency of power, in the current study we performed a meta-analysis of the independent RNA-seq datasets publicly available. We performed gene differential expression meta-analysis of RE+ cases vs. controls and RE- cases vs. controls, separately, and compared the similarities and differences of the transcriptomic profile changes in terms of biological pathways. To identify biological processes and functions orchestrated by genes with similar expression patterns, we also constructed co-expression networks for RE+ and RE- patients, separately, and made quantitative comparisons. The gene differential expression and co-expression meta-analysis results provide more extensive and less biased results to help understand the molecular mechanisms of FECD.

## Methods

### Datasets

We performed a transcriptomic meta-analysis of FECD based on RNA-seq datasets of human corneal endothelial tissues. Our group previously generated a dataset with 6 RE+ cases, 4 RE- cases, and 9 controls, which was accessible at Gene Expression Omnibus (GEO; GSE142538) [18]. It is denoted as dataset I throughout the paper. We also queried PubMed, the European Nucleotide Archive, and the Sequence Read Archive (SRA) for the inclusive terms “eye”, “cornea”, and “RNA-seq”, and identified two more datasets. One was from SRA with accession number PRJNA524323 [26] including 8 RE+ cases, 4 RE- cases, and 6 controls. It is denoted as dataset II. The other was from GEO with accession number GSE112201 [22], which is denoted as dataset III. Note that there were three batches in this dataset but only batch 2 contained control samples. To minimize unknown confounding effects we only used the batch 2 data. We also omitted the only RE- sample in the batch, resulting a total 11 RE+ cases and 4 controls considered for meta-analysis from this dataset. To sum, there were a total of 25 RE+ cases, 8 RE- cases, and 19 controls included in the meta-analysis (Table 1). Note that controls did not carry the CTG18.1 expansions. The Preferred Reporting Items for Systematic Reviews and Meta-Analyses (PRISMA) flow diagram is presented in S1 Fig.

**Table 1:** Fuchs’ endothelial corneal dystrophy RNA sequencing datasets for meta-analysis.

Study	Phenotype	Genotype	Sample size	Read length (bp)
I GEO: GSE142538	case	RE+	6	150
		RE-	4	
	control	RE-	9	
II SRA: PRJNA524323	case	RE+	8	125
		RE-	4	
	control	RE-	6	
III GEO: GSE112201	case	RE+	11	101
	control	RE-	4	

Genotype RE+ and RE- refer to without and without the CTG18.1 expansion allele (≥40 repeats). All samples were sequenced with paired-end reads on an Illumina platform.

All raw datasets are publicly available. We share all the detailed results in the Supplementary materials. Intermediate files can be requested from the corresponding authors.

### Differential expression and alternative splicing analyses

Raw RNA-seq datasets were downloaded using the NCBI SRA toolkit (https://github.com/ncbi/sra-tools). Sequencing reads were aligned to human hg38 reference genome using STAR (v.2.5.3a) [27], and counted using featureCounts (v.1.4.6) [28], with gene symbols labeled according to the UCSC human genome hg38 GTF file (https://support.illumina.com/sequencing/sequencing_software/igenome.html). Differential expression analysis was first performed within each dataset using edgeR (v.3.18.1) [29]. Meta-analysis of RE+ cases vs. controls and RE- cases vs. controls was then conducted, separately, using AWFisher (v.1.8.0) [30], which integrates the p-values from individual studies by an adaptively weighted Fisher’s method and reports the false discovery rate (FDR) adjusted p-values. In a single study, genes with FDR < 0.05 were considered as differentially expressed genes (DEGs); in meta-analysis, we took a conservative approach that genes with FDR < 0.05 and a consistent change direction among individual studies were regarded as DEGs. Differential alternative splicing analyses of RE+ cases vs. controls and RE- cases vs. controls were performed within each dataset using rMATS turbo (v4.1.2) [31]. The splicing events with FDR < 0.05 and > 5% change in the inclusion levels were considered differentially spliced. Venn diagrams were drawn with VennDiagram (v1.7.3) [32]; UpSet plots were drawn with UpSetR (v1.4.0) [33].

### Gene co-expression network analyses

Besides the marginal differential expression analysis, we further performed gene co-expression analysis to identify biological processes and functions orchestrated by genes with similar expression patterns. In general, the analysis involves three steps [34]: (1) to calculate pairwise similarity measures such as Pearson’s correlation between genes based on the gene expression levels in a group of samples; (2) to construct a gene co-expression network with each node representing a gene and each edge representing the strength of correlation, as calculated in (1) between two genes; (3) to apply clustering algorithms to trim the massive network, as constructed in (2), into clusters of highly connected genes with close co-expression patterns. A cluster of highly connected genes constitute a module, and genes with high connectivity in the module are called hub genes, which can serve as representatives of a module. Subsequently modules can be interpreted by functional enrichment analysis etc.

In the current analysis we performed gene co-expression analysis by using the weighted gene co-expression network analysis (WGCNA) package (v.1.71) [35, 36]. Gene co-expression networks were constructed for RE+ and RE- cases, separately, by integrating relevant samples from all studies. First, batch effects were adjusted by using the ComBat-seq method implemented in the R package sva (v.3.42.0) [37, 38]. The control samples in each study were also included at this step to best adjust for the batch effects. Next, genes with adjusted counts >10 in more than 90% of the samples were kept, and transcripts per million were calculated [39, 40]. Third, genes with top 1000 median absolute deviations were selected to construct the undirected gene co-expression networks using WGCNA. The signed correlation network adjacencies were calculated with certain soft-thresholding power to scale the network and obtain the topologically overlapped matrix (TOM) [41]. Network modules were identified with a dynamic tree cut algorithm [42]. The resulting transcriptomic modules were highly intra-connected gene clusters based on similar gene expression profiles, except that the grey module contained genes unable to constitute modules and would not be further considered.

Upon building the separate RE+ and RE- gene co-expression networks, we updated the networks by comparing the correlation of modules within and between networks and merging modules within each network iteratively using the following steps. (1) A matrix of Jaccard indices among modules with each row / column corresponding to an RE+ / RE- module is calculated. For each matrix element Fisher’s exact test is also performed to assess the significance of gene overlapping between two modules. (2) The rows and columns of the matrix are rearranged by putting the highest Jaccard index to the diagonal recursively. (3) If all elements of a diagonal block are with Fisher’s exact test p-value <0.05 and the modules are originated from the same branch in their respective clustering trees, the modules are merged. (4) If two rows / columns of one network show similar correlation patterns with columns / rows of the other network, and the modules are originated from the same branch of the clustering trees, the modules are merged. (5) Repeat steps (1)-(4) until the modules in each network cannot be further merged. By this algorithm, we were able to construct RE+ and RE- networks with similar and distinct modules based on the extent of overlapping genes.

For the paired modules between RE+ and RE- networks, we further investigated the extent they were preserved by comparing their topological structure summarized by the TOM of hub genes. The hub genes were those with top 25% signed eigengene-based connectivity in each module [35, 43]. The null hypothesis of network comparison is non-preservation. Module preservation statistics [44] were calculated based on a network permutation procedure [45], and it is recommended not to reject the null if the summary statistic *z_sum_* < 2 [46].

### Biological pathway analyses

The functions of gene sets, either DEGs or genes in a co-expression module, were investigated by pathway enrichment analysis using WebGestalt [47]. Both over-representation analysis (ORA), based on a short list of selected genes, and gene-set enrichment analysis (GSEA) [48, 49], based on the ranking of a relatively long gene list, were performed whenever appropriate. Pathways and biological processes with FDR < 0.05 were considered significantly enriched. To identify the transcriptional regulators that can potentially explain the observed gene expression changes, we also performed upstream regulator analysis (URA) based on the meta-analysis results of differential gene expression using Ingenuity Pathway Analysis (IPA) software [50].

## Results

### Differential expression analyses

**Fig 1:**
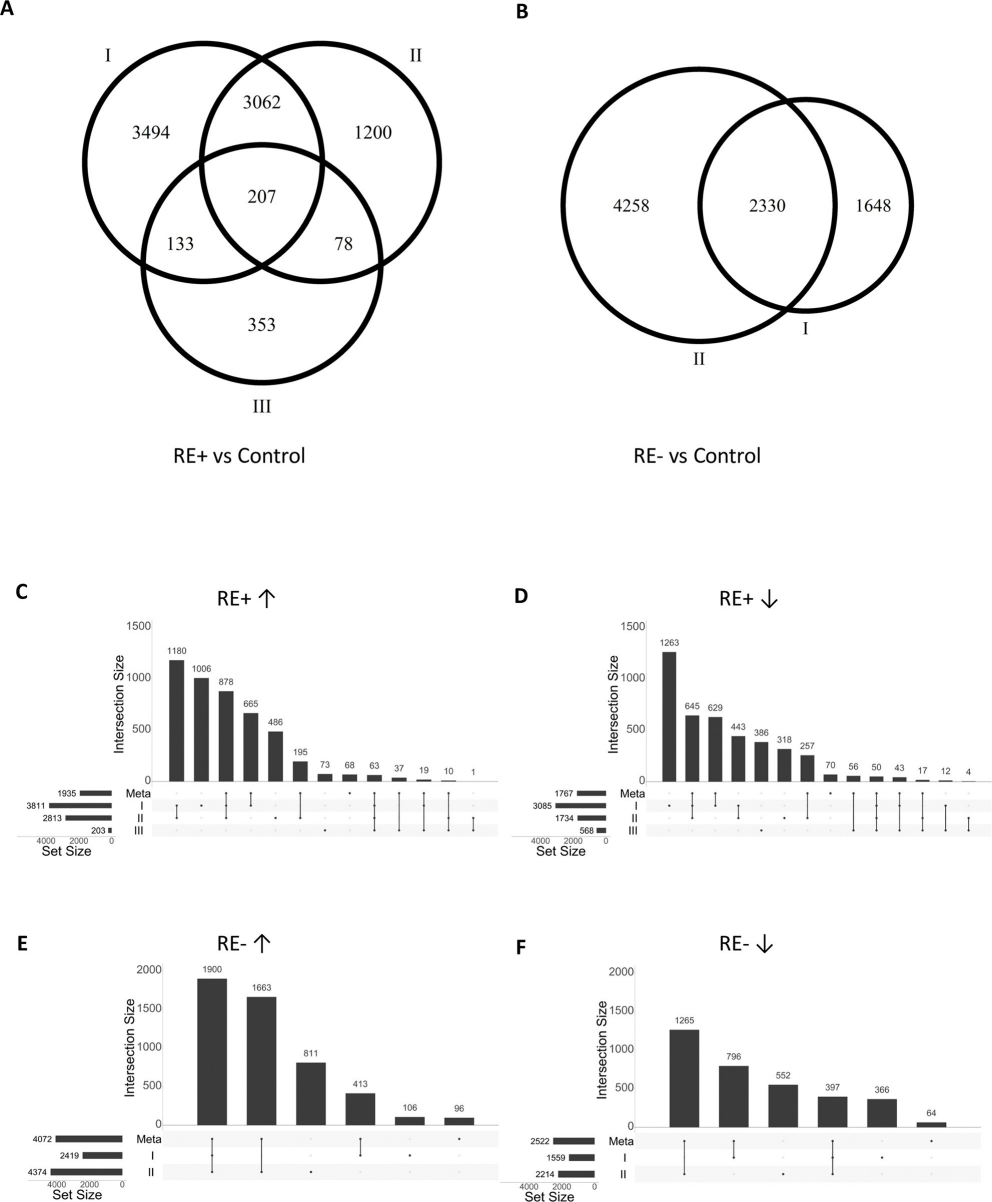
Fuchs’ endothelial corneal dystrophy (FECD) differential gene expression analysis by single studies and meta-analysis. (A) and (B): Venn diagrams of differentially expressed genes (DEGs) between FECD patients with the *TCF4* CTG18.1 expansion (RE+) versus controls and patients without the expansion (RE-) versus controls from single studies. (C)—(D): UpSet plots of DEGs in each single study and by meta-analysis. Arrows ↑ and ↓ represent up- and down- regulated genes, respectively. Datasets I, II, and III refer to GEO: GSE142538, SRA: PRJNA524323, and GEO: GSE112201, respectively. In a single study, genes with the false discovery rate (FDR) < 0.05 were considered as DEGs; in meta-analysis, genes with FDR < 0.05 and a consistent change direction among individual studies were regarded as DEGs.

**Fig 2:**
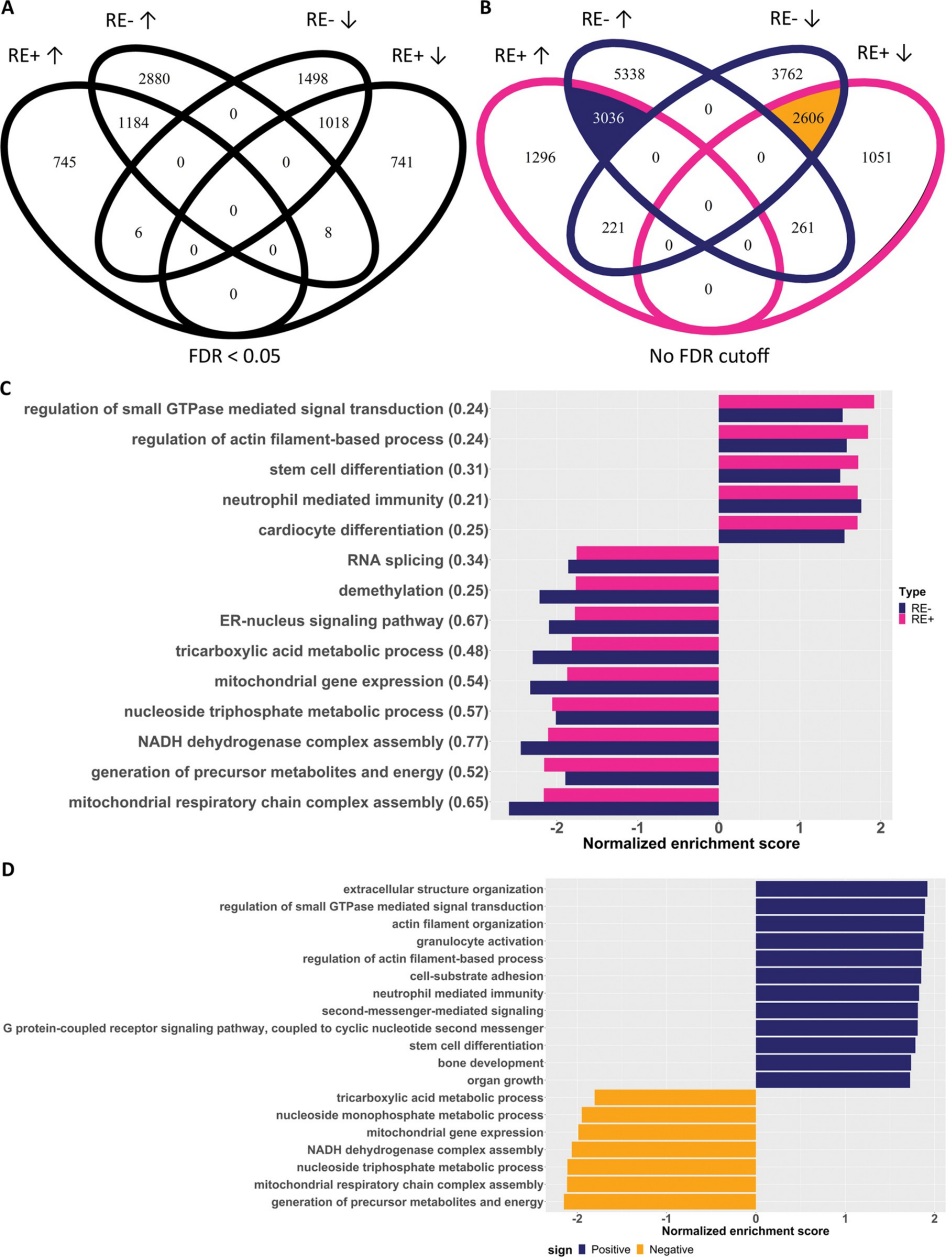
Comparison of the differentially expressed genes (DEGs) in meta-analysis between the two Fuchs’ endothelial corneal dystrophy (FECD) types. (A): Venn diagrams of DEGs of four categories of DEGs. RE+ and RE- refer to FECD patients with and without the *TCF4* CTG18.1 expansion, respectively; arrows ↑ and ↓ represent up- and down-regulated genes, respectively. Genes with the false discovery rate (FDR) < 0.05 and a consistent change direction among individual studies were regarded as DEGs. (B) Venn diagrams of genes with a consistent change direction among individual studies, i.e., lifting the FDR criterion compared to (A). (C) The significantly enriched biological processes (FDR < 0.05) shared between RE+ and RE- transcriptomes by gene set enrichment analysis (GSEA). The Jaccard index of the enriched genes for each process between the two groups was shown in the parenthesis. (D) The significantly enriched biological processes (FDR < 0.05) by the shared genes between RE+ and RE- transcriptomes (those in the shaded area of B).

There was a large difference among studies in terms of the number of DEGs (Fig 1A and 1B), which manifested the potential limitations of making inferences based on single studies. Intersecting the DEGs indicated the weights of each study to the meta-analysis results (Fig 1C–1F). For comparison between RE+ cases and controls, dataset I weighted the most with more than 1/3 (665/1935 and 629/1767 for up- and down-regulated genes, respectively) of DEGs in meta-analysis being significant only in dataset I; dataset III weighted the least. For comparison between RE- cases and controls, dataset II weighted more than dataset I. Note that for RE+ vs. controls there were less than 5% (63/1935 and 50/1767 for up- and down-regulated genes, respectively) DEGs being significant in all the three studies, which suggests the conventional practice of using overlapping signals lead to loss of power. On the contrary, there were many DEGs in meta-analysis that were insignificant in any single study, which highlighted the power gain of meta-analysis. Take a few examples—*HK2*, *PFKFB3* and *SNCA* were involved in the nucleoside triphosphate metabolic process, a biological process negatively enriched in both RE+ and RE- transcriptomes (Fig 2C and 2D); a cluster of ribosome proteins *RPL14*, *RPL27*, *RPL35A*, *RPLP2*, *RPS12*, *RPS14*, and *RPS18* were involved in the translational initiation process, which was negatively enriched in the RE+ transcriptomes (S1 Table). They were all downregulated in the three individual RE+ studies, but none reached statistical significance in a single study. However, they met the FDR < 0.05 criterion in the meta-analysis.

The transcriptomic profiles of RE+ and RE- cases showed a great extent of similarity based on the meta-analysis DEGs compared to controls (S2 Table). There were 1,184 genes significantly upregulated and 1,018 genes significantly downregulated in both RE+ and RE- cases; in contrast, there were only 6 genes upregulated in RE+ but downregulated in RE- cases, and 8 vice versa (Fig 2A). The transcriptomic similarity held when the FDR criterion was lifted, i.e., only requiring a consistent change direction among individual studies (Fig 1B). Note that there were more DEGs or genes with a consistent change direction in RE-. We suspect it was because there were only two datasets in RE- to filter for genes with the consistent change direction.

**Fig 3:**
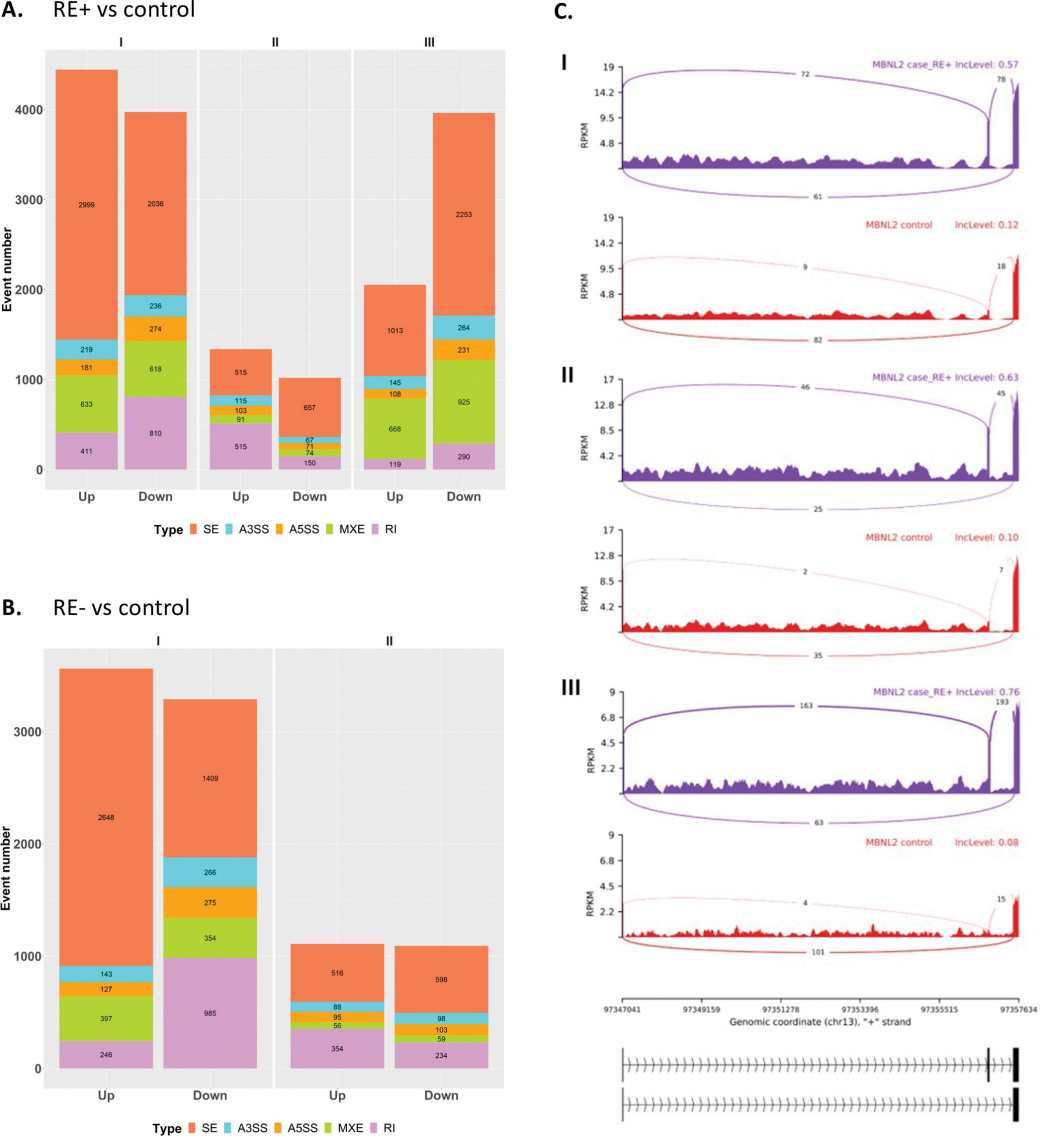
Differential alternative splicing events in Fuchs’ endothelial corneal dystrophy (FECD) patients. Numbers of the 5 types of differential alternative splicing events were plotted in RE+ (A) and RE- (B) in patients compared with controls in each dataset (I, II, and III). Events with false discovery rate < 0.05 and > 5% changes in the inclusion levels were selected. “Up” and “Down” refer to upregulated and downregulated events compared to control, respectively. SE: skipped exon; A3SS: alternative 3’ splice site; A5SS: alternative 5’ splice site; MXE: mutually exclusive exons; RI: retained intron. The Sashimi plots (C) depicted an SE event in *MBNL2* that was observed in all the 3 individual studies of RE+ vs control. The average inclusion level of each group (the percentages of the transcripts with the alternative exon) was labelled at the upper right corner. The y-axis indicated the average read density measured by fragments per kilobase of transcript per million (FPKM), and the average number of junction-spanning reads were labeled on the curves. Human hg38 genomic coordinates and relevant exons were drawn below.

Pathway analysis was performed to investigate the molecular pathways involved in FECD. First, analyses for RE+ and RE- cases were carried out separately, and the results were compared (S3 Table). To maximize the power of GSEA, we used the gene lists with the FDR criterion lifted. There were 14 biological processes significantly enriched in both RE+ and RE- cases and they were all in a consistent enrichment direction between the two conditions (Fig 2C). Among the shared processes, mitochondrial functions, energy-related processes, ER-nucleus signaling pathway, demethylation, and RNA splicing were negatively enriched; small GTPase mediated signaling, actin-filament processes, stem cell differentiation, neutrophil mediated immunity, and cardiac cell differentiation were positively regulated. The Jaccard index of the enriched genes for each process between the two groups was calculated. The processes related to mitochondria, energy, and ER-nucleus signaling had higher overlaps, indicating that the two types of FECD might share similar molecular mechanisms in mitochondrial dysfunction and ER-nucleus signaling pathway. Note that the RNA splicing process was negatively enriched in both RE+ and RE- cases. Differential alternative splicing analysis identified predominantly exon skipping events in all the comparisons (Fig 3A and 3B).

Given the similarity of RE+ and RE- transcriptomic profiles, we further performed GSEA based on the shared genes between the two conditions (3036 + 2606 in Fig 2B), aiming to identify the most conserved pathways that were perturbed in FECD compared to controls (Fig 2D and S4 Table). The gene list was ranked based on the combined p-values of RE+ and RE- meta-analysis p-values using Fisher’s method. Note that all the negatively enriched biological processes were related to mitochondrial and energy metabolism. On the positively enriched biological processes, besides actin-related processes, immunity, stem cell differentiation, and cell signaling pathways, processes involving extracellular structure organization and cell adhesion also showed up.

### Gene co-expression network analyses

**Fig 4:**
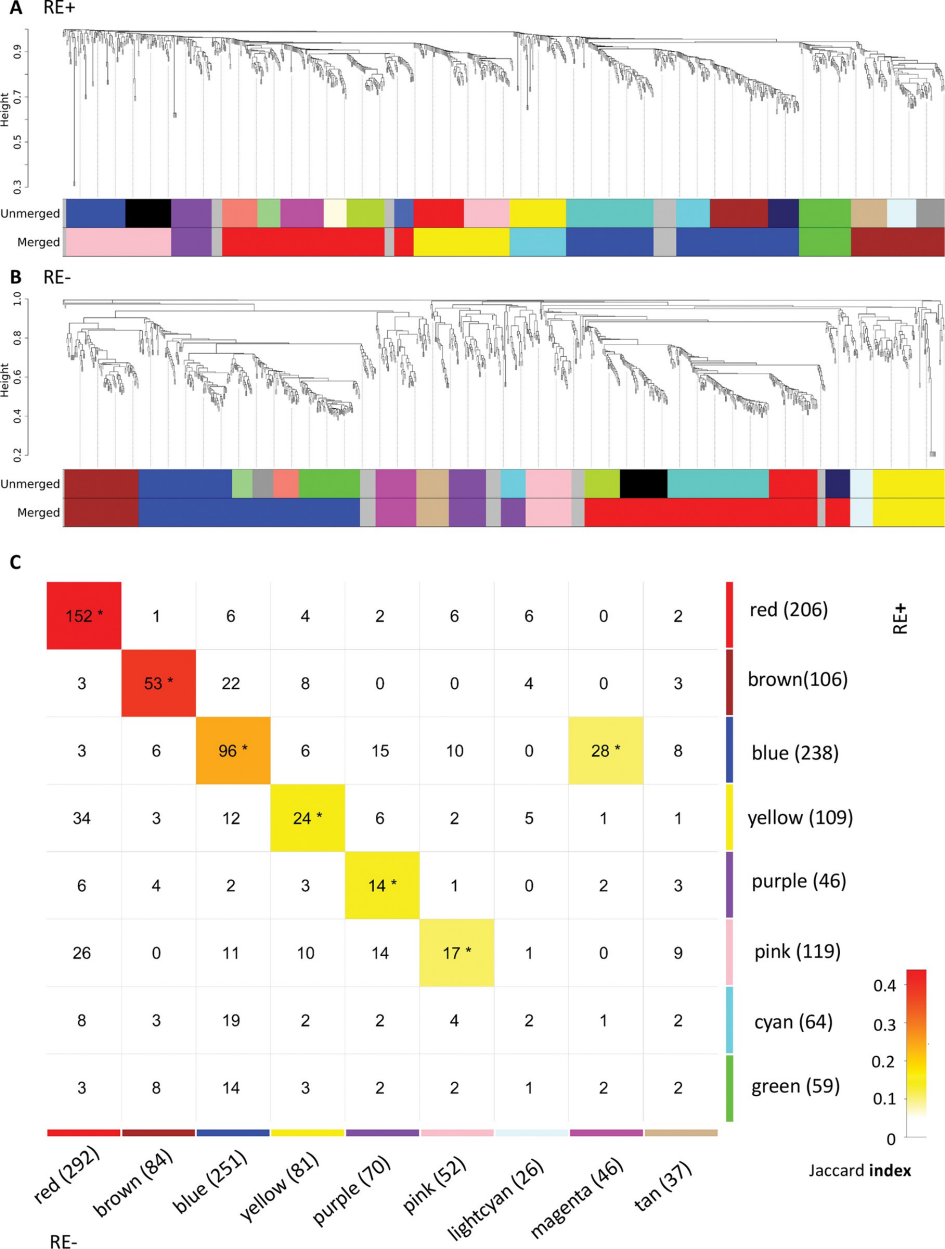
Gene co-expression networks in Fuchs’ endothelial corneal dystrophy (FECD) patients with (RE+) and without (RE-) the *TCF4* CTG18.1 expansion. (A) and (B): Hierarchical clustering dendrograms based on genes with top 1000 median absolute deviations for RE+ and RE- transcriptomic profiles, respectively. Different color represents distinct modules. The top and bottom bars indicate modules before and after merging. (C): Correlations between RE+ and RE- modules after merging. The number of genes in each module were shown after the module name in the parenthesis. In each block, the number showed the number of intersected genes. * indicated module pairs with significant correlation (false discovery rate < 0.05). The color indicated the Jaccard index, and was left blank (value = 0) if the correlation was not significant.

The gene co-expression networks for RE+ and RE- profiles were first built separately and then updated by merging modules through borrowing information from each other (Fig 4). In the end there were 8 modules with the number of genes ranging from 46 to 238 in each module for the RE+ profiles, and 9 modules with the number of genes ranging from 26 to 292 for the RE- profiles (S5 Table). There were 6 modules (red, brown, blue, yellow, purple, and pink) showing significant similarity in terms of constituted genes between RE+ and RE- profiles; in addition, the magenta module in RE- was also significantly correlated with the blue module in RE+. Pathway analysis of the overlapping genes between the paired modules revealed their common functionality (Table 2 and S6 Table). Modules of different color showed relatively distinct biological processes enriched, which justifies the use of gene co-expression networks to investigate the interactive molecular mechanisms. The cyan and green modules in RE+ and lightcyan and tan modules in RE- did not have obvious counterparts in the other FECD type. Pathway analysis results showed related biological processes that were also enriched in some shared modules, e.g., immunity and extracellular matrix organization.

**Table 2:** Shared modules of gene co-expression networks in Fuchs’ endothelial corneal dystrophy (FECD) patients with (RE+) and without (RE-) the *TCF4* CTG18.1 expansion.

Modules		# of genes	Enriched biological processes*	Shared differentially expressed genes		
RE+	RE-			Counts	Direction	Names
red	red	152	mitochondrial respiratory chain complex assembly	77	down	*COX5B;NDUFB9;NDUFA13;NDUFA1;NDUFB2;NDUFAB1;NDUFB5;COX20; NDUFV1;NDUFB8;NDUFS1;MDH1; SDHB;SDHD;OGDHL* **
			tricarboxylic acid metabolic process			
			nucleoside triphosphate metabolic process			
brown	brown	53	translational initiation	4	down	*NME1-NME2;NME2;MIR4426;RPL34*
			ribonucleoprotein complex biogenesis			
			translational elongation			
blue	blue	96	podosome assembly	12	up	*SPARC;SERPINE2;TAGLN2;GNS; LOXL1;MIR198;COL1A2;FSTL1; PALLD;GAS1;MAGED1;PLD3*
			response to interleukin-12			
			regulation of defense response to virus by virus	3	down	*JUNB;SOD2;MCL1*
purple	purple	14	adaptive thermogenesis	5	down	*VEGFA;SNORD2;CCNL1;RBM39;IDI1*
			RNA splicing			
yellow	yellow	24	—	2	down	*SERP1;SRP9*
pink	pink	17	—	1	down	*ERRFI1*
blue	magenta	28	—	3	up	*IGFBP2;PAMR1;MDK*

*based on over-representation analysis of genes in the former column; similar processes were merged and the top 3 processes were listed with detailed results in S6 Table;—denotes no enriched process.

**Only 15 genes are listed here. All 77 genes are documented in S6 Table.

**Fig 5:**
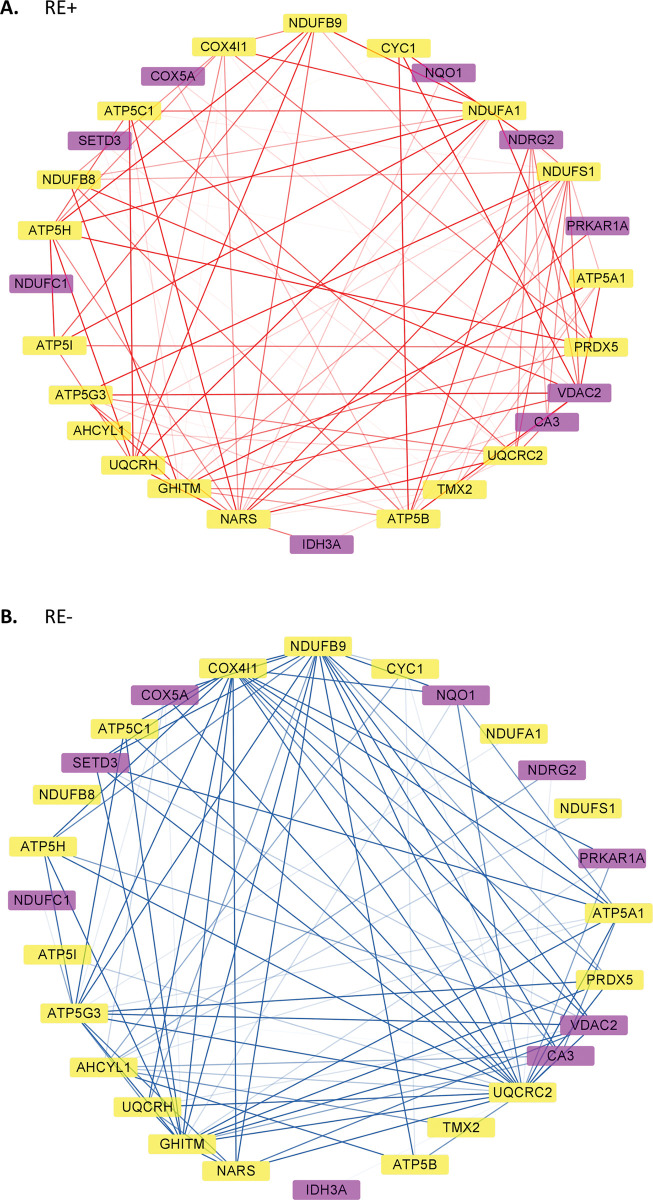
Comparison of the red modules in Fuchs’ endothelial corneal dystrophy (FECD) patients with (RE+) and without (RE-) the *TCF4* CTG18.1 expansion. (A) and (B): The subnetworks of 28 shared hub genes in the red module for RE+ and RE- transcriptomic profiles, respectively. The hub genes were defined as those with top 25% intramodular connectivity. Deeper color of edges indicated higher connectivity values. Edges with top 25% weights in the subnetwork were shown. Color yellow: genes significantly downregulated in both RE+ and RE-; purple: genes significantly downregulated only in RE-. A permutation test indicated the network was moderately preserved (*z_sum_* = 2.14).

Among the 152 shared genes in the red module, more than half (77) are DEGs in both RE+ and RE- cases vs. controls. It showed the most common feature of RE+ and RE- transcriptomic changes compared to controls was the downregulation of genes in mitochondrial functions and metabolism-related processes, which were enriched with the red module genes. We further investigate whether there were topological differences between RE+ and RE- red modules by focusing on the 28 genes that were hub genes in both modules (Fig 5). A permutation test indicated that the network was moderately preserved (*z_sum_* = 2.14). Among the 28 genes, 19 (*AHCYL1*, *ATP5A1*, *ATP5B*, *ATP5C1*, *ATP5G3*, *ATP5H*, *ATP5I*, *COX4I1*, *CYC1*, *GHITM*, *NARS*, *NDUFA1*, *NDUFB8*, *NDUFB9*, *NDUFS1*, *PRDX5*, *TMX2*, *UQCRC2*, *UQCRH*) were DEGs in both RE+ and RE- cases, and 9 (*CA3*, *COX5A*, *IDH3A*, *NDRG2*, *NDUFC1*, *NQO1*, *PRKAR1A*, *SETD3*, *VDAC2*) were DEGs only in RE-. Note that many of these hub genes are related to oxidative phosphorylation (OXPHOS), the process by which electron transport chain and chemiosmosis are utilized in the mitochondria to produce adenosine triphosphate (ATP).

### Genes related to oxidative phosphorylation

**Fig 6:**
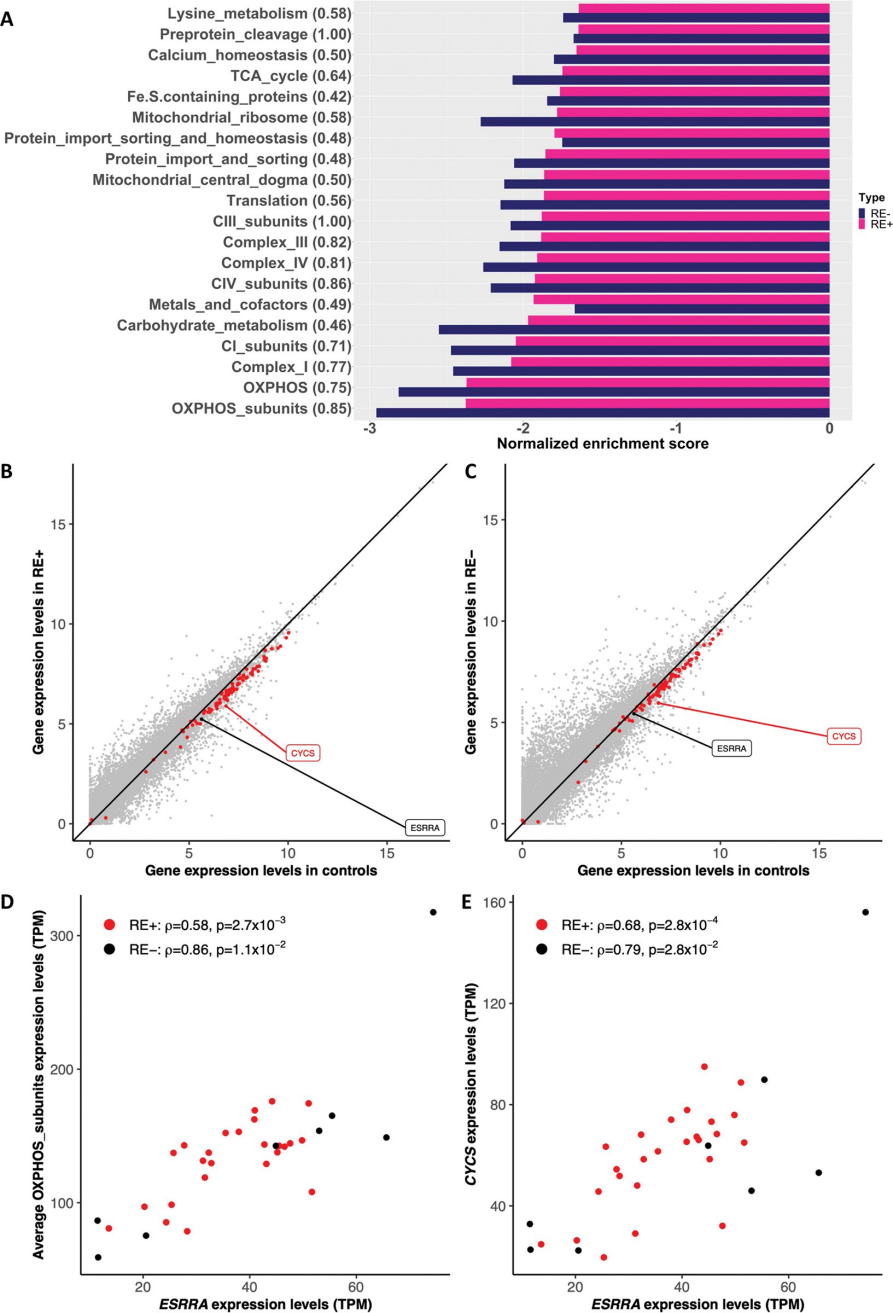
Dysregulation of mitochondrial pathways in Fuchs’ endothelial corneal dystrophy (FECD) patients with (RE+) and without (RE-) the *TCF4* CTG18.1 expansion. (A) The significantly enriched mitochondrial pathways shared between RE+ and RE- transcriptomes by gene set enrichment analysis (false discovery rate < 0.05). The Jaccard index of the enriched genes for each process between the two groups was shown in the parenthesis. The mean expression levels of genes in controls were plotted against that in RE+ (B) and RE- (C), respectively. The unit is ${log}_{2}^{\left(TPM+1\right)}$, where TPM denotes transcripts per million. The red dots were oxidative phosphorylation OXPHOS subunit genes assayed (n = 88). Two genes—estrogen-related receptor alpha (*ESRRA*) and cytochrome c (*CYCS*)—were highlighted. The expression levels of *ESRRA* were correlated with the average expression levels of OXPHOS subunit genes (D) and with the expression levels of *CYCS* (E) in both RE+ (red) and RE- (black) patients.

The differential expression analyses and gene co-expression network analyses highlighted mitochondrial and energy metabolism processes in both RE+ and RE- cases. Therefore, we performed GSEA of MitoPathways3.0 [51], which consists of 149 hierarchical pathways with 1136 mitochondrial proteins to better understand mitochondrial dysfunction in FECD. There were 394 and 366 DEGs in RE+ and RE-, respectively, among which 288 were shared. There was great extent of similarity on the mitochondrial pathways involved, and all were negatively enriched (Fig 6A and S7 Table). A majority of these mitochondrial pathways are related to OXPHOS. Consistently, the expression levels of OXPHOS subunit genes were universally downregulated in both RE+ and RE- cases (Fig 6B and 6C).

It is known that ERRα is a key regulator of OXPHOS [52]. Indeed, the URA identified *ESRRA* as a significant upstream regulator in both RE+ and RE- cases (S8 Table). Moreover, *ESRRA* was significantly downregulated in both RE+ and RE- cases (FDR = 9.0×10^−4^ and 9.6×10^−3^ in RE+ and RE-, respectively; S2 Table). We hypothesized that the expression level of *ESRRA* was correlated with that of OXPHOS subunit genes. Of the 88 OXPHOS subunit genes assayed, 53 and 60 were significantly correlated (P-value of Spearman’s correlation < 0.05) with *ESRRA* expression levels in RE+ and RE-, respectively, and 49 genes were in common (S9 Table). The correlations of *ESRRA* with the average expression levels of OXPHOS subunit genes and with a representative gene, cytochrome c, were shown (Fig 6D and 6E). Collectively, these data suggest that ERRα orchestrates OXPHOS in FECD tissue.

ERRα regulates the transcription of metabolic genes through multiple pathways such as oxidative stress and autophagy. There were 43 direct target genes of ERRα that were significantly changed in the same direction in both RE+ and RE- patients according to the Ingenuity Knowledge Base [50] (S10 Table). We performed ORA on these 43 genes to identify other pathways potentially regulated by ERRα. There were 60 biological processes significantly enriched (FDR<0.05) including angiogenesis, response to oxygen levels, and response to oxidative stress.

## Discussion

Characterizing the transcriptomic profile changes of disease tissues is crucial to understanding pathophysiological pathways involved, identifying biomarkers to classify disease types and predict disease progression, and developing prevention and therapeutic strategies. In this study we performed a meta-analysis of late-onset FECD RNA-seq datasets, aiming to identify similarities and differences of the transcriptomic profile changes between RE+ and RE- FECD. Meta-analysis synthesizes data from multiple independent studies by quantitative methods to reach conclusions [53, 54]. It can achieve more power by integrating supporting evidence from multiple studies and thus preventing premature dismissal of potential signals from small underpowered studies. In the current study, for example, the meta-analysis identified 298 DEGs that were insignificant in any single study but were in consistent change direction across studies (S1 Table and Fig 1C–1F). It can also reduce bias dominated by single studies. Take an example, there were 94 genes with FDR < 0.05 in each of the three studies of RE+ vs control but fold change in opposite directions between studies. One would make opposite inferences on these genes depending on which study to base on. In contrast, none of them were DEGs by meta-analysis.

To date, there is no report on clinically distinguishing RE+ and RE- late-onset FECD; consistently, their transcriptomic profiles showed high similarity in terms of both differential expression compared to controls and gene co-expression networks. The hallmark of FECD transcriptomic profiles was the downregulation of genes in pathways related to mitochondrial functions and energy metabolism, which is no surprise as FECD is an age-related degenerative disease [55]. In order to maintain deturgescence for the transparency of cornea, corneal endothelial cells regulate the osmotic force through the active transport of ions, which highly relies on the energy from mitochondria [56], and abnormal mitochondria have been proposed to play an important role in FECD (for a review see, e.g., reference [57]). Examination of the hierarchical pathways related to mitochondria [51] revealed that defects of OXPHOS are predominant in the transcriptional signature of FECD in both RE+ and RE- cases. Further, we showed the expression level of *ESRRA* was correlated with that of OXPHOS subunit genes, which is consistent with the knowledge that ERRα is an upstream regulator of OXPHOS [52]. ERRα modulates genes related to mitochondrial biogenesis and OXPHOS in the skeletal muscle of patients with diabetes mellitus [48, 58] and the myocardium of patients with failing hearts [59]. It is of interest to note that an age-related decrease in OXPHOS gene expression has been observed in brain tissue [60], cardiac muscle [61], and skeletal muscle [62], as FECD is also an age-related disorder.

ERRα regulates the transcription of metabolic genes in multiple biological processes [63–65]; consistently, many processes were enriched in the ERRα target genes that were differentially expressed in FECD (S10 Table). The enrichment of response to oxidative stress pathway agrees with the observation of oxidant-antioxidant imbalance and accumulation of oxidized DNA in the corneal endothelium of FECD patients [66]. ERRα was reported to be involved in hypoxia response and angiogenesis [65]; consistently these was an enrichment of biological processes angiogenesis and response to oxygen levels. The enrichment of pathways like pyruvate metabolic process and regulation of lipid metabolic process confirms its involvement in regulating glucose and lipid metabolism. ERRα is also a key coordinator of autophagy [67]; however, this process does not seem to be involved in FECD as none of the autophagy-related genes listed in reference [67] were consistently differentially expressed.

It is of interest to note that the RNA splicing process was negatively enriched in both RE+ and RE- cases. Consistently, there were only slight smaller amounts of alternative splicing events in RE- than in RE+ cases (Fig 3A and 3B). Note that there were much smaller numbers of alternative splicing events in dataset II for both RE+ and RE- cases, which might be related to the sequencing library preparation protocol. A working hypothesis for the RE+ cases is that the CUG repeat expansion on *TCF4* RNA sequesters critical RNA splicing factors, leading to broad dysregulation of RNA splicing [18, 23, 24]. However, the dysregulation of RNA splicing process in the RE- cases remains unexplained. One hypothesis is that repeat expansions in other genes cause FECD, as the CTG expansion within the 3′-untranslated region of *DMPK* does [13, 19]. There is also a likelihood that mechanisms other than toxic RNA lead to a proportion of RE- FECD. Supportive of this notion is that an increase of the inclusion levels of an *MBNL2* exon was observed in the RE+ cases of all three studies (Fig 3C), but not in RE- cases.

Although the RE+ and RE- transcriptomic profiles were alike to large extent, there were detectable differences that might be due to the underlying the molecular mechanism. The translational initiation process was enriched with genes in the brown module pairs; however, when performing pathway analysis based on genes with different expression levels, it was only enriched in RE+ but not in RE- profiles (S3 Table). Of the 31 leading-edge genes of GSEA that were also in the brown module, 26 were downregulated with FDR < 0.05 in RE+ compared to controls, including the 7 ribosome proteins previously mentioned to meet the criterion only in the meta-analysis; in contrast, only 1 was with FDR < 0.05 in RE-. It suggested a possible change in the ribosomal biogenesis in RE+ different from RE-. In the literature it was shown perturbation of *TCF4* could disrupt ribosomal biogenesis—in a *TCF4* knockdown cell line the ribosome biogenesis processes were enriched in downregulated genes including *RPL35A* [68]; overexpressing dominant-negative *TCF4* mutant in cultured hippocampal neuron led to reduced perikaryal ribosome numbers and protein synthesis [69]. Thus we hypothesize that aberrant *TCF4* transcripts in the RE+ patients could lead to down-regulation of genes encoding ribosomal constitution-related proteins. There were 221 genes consistently upregulated in each single study of RE+ but downregulated in each study of RE-, and there were 261 genes vice versa (Fig 2B), which could be due to different molecular mechanisms of RE+ and RE- FECD. However, we do not have a plausible interpretation of the enriched pathways (S11 Table).

A major limitation of the current study is still the small sample size, though it is already an attempt of meta-analysis. In our previous analysis of dataset I [18] we were able to determine that upregulation of extracellular matrix and fibrosis genes, activation of the immune system, and mitochondrial dysfunction are common final molecular processes of late-stage FECD. However, the defects in mitochondrial dysfunction and OXPHOS were not as readily apparent in RE- cases as in the current meta-analysis study, which further exemplified the power of meta-analysis. We look forward to integrating new RNA-seq datasets whenever available by the same workflow to provide more comprehensive and less biased view of FECD in future. We did not include microarray expression datasets because of less sensitivity and the numbers of genes assayed different from that of RNA-seq datasets. Another limitation is that the tissues were from advanced FECD patients. The overwhelming similarity of RE+ and RE- transcriptomic profiles might be partially confounded by the degenerative nature of the disease. Caution needs to be taken to when interpreting the results as causes or consequences.

## Supporting information

S1 Fig The Preferred Reporting Items for Systematic Reviews and Meta-Analyses (PRISMA) flow diagram. (TIF)

S1 Table Differentially expressed genes (DEGs) identified by meta-analysis but not by any single study. Genes in this table have a consistent change direction among individual studies with FDR > 0.05 in each single study but with FDR < 0.05 by meta-analysis.
(XLSX)

S2 Table The overall differential expression results of meta-analysis results. This table contains the detailed data of Fig 2A and 2B. Genes with a consistent direction change of expression levels among individual studies of RE+ or RE- were marked in the columns with “direction”; an empty cell denotes inconsistent change directions.
(XLSX)

S3 Table Gene set enrichment analysis (GSEA) of genes with a consistent direction change of expression levels among individual studies of RE+ or RE-. Sheet 1 contains the shared biological processes; sheets 2 and 3 contain RE+ and RE- enriched processes, respectively. The column “leading edge genes” refers to the genes maximizing the enrichment scores in the GSEA.
(XLSX)

S4 Table Gene set enrichment analysis (GSEA) of the shared genes with a consistent direction change of expression levels among individual studies of RE+ or RE-. Sheet 1 contains the enriched biological processes; sheet 2 details the leading edge genes of each enriched process. A cell marked with “X” indicates “Yes” for the column.
(XLSX)

S5 Table Gene co-expression networks of RE+ and RE- transcriptomes. This table lists the differential expression results and co-expression network modules of the genes. Genes with a consistent direction change of expression levels among individual studies of RE+ or RE- were marked in the columns with “sign”; an empty cell denotes inconsistent change directions. Column “moduleHub” indicates whether a gene is a hub gene of a module. Each module has a designated column. A cell marked with “X” indicates “Yes” for the column.
(XLSX)

S6 Table Shared modules of gene co-expression networks of RE+ or RE- and their enriched biological processes. Sheet 1 contains the summary information; sheets 2 to 5 contain pathway analysis results for red, brown, blue, and purple module pairs, respectively.
(XLSX)

S7 Table Gene set enrichment analysis (GSEA) of MitoCarta genes with a consistent direction change of expression levels among individual studies of RE+ or RE-. Sheet 1 contains the shared biological processes; sheets 2 and 3 contain RE+ and RE- enriched processes, respectively.
(XLSX)

S8 Table Differentially expressed upstream regulators shared between RE+ and RE-. The target genes of the upstream regulators were listed for RE+ and RE-, respectively, with the shared targets listed in a separate column.
(XLSX)

S9 Table Spearman’s correlation between the expression levels of ESRRA and OXPHOS subunits genes. There were 88 oxidative phosphorylation (OXPHOS) subunit genes assayed.
(XLSX)

S10 Table ERRα targets with a consistent direction change of expression levels between the meta-analyses of RE+ and RE-. Sheet 1 details the ERRα target genes significantly changed in the same directions in both RE+ and RE-; sheet 2 contains the enriched biological processes of the target genes (FDR<0.05).
(XLSX)

S11 Table Over-representation analysis (ORA) of genes with an opposite direction change of expression levels between RE+ and RE-. Enriched pathways (FDR < 0.05) of 487 genes with consistent fold change directions among replicates of either RE+ or RE-, but with opposite directions between RE+ or RE-, as shown in Fig 2B.
(XLSX)
