# Supplementary figures and images for: Transcriptomic meta-analysis reveals ERRα-mediated oxidative phosphorylation is downregulated in Fuchs’ endothelial corneal dystrophy
Source: PLoS One. 2023 Dec 14;18(12):e0295542. doi: 10.1371/journal.pone.0295542 (PMC10721014; doi:10.1371/journal.pone.0295542)

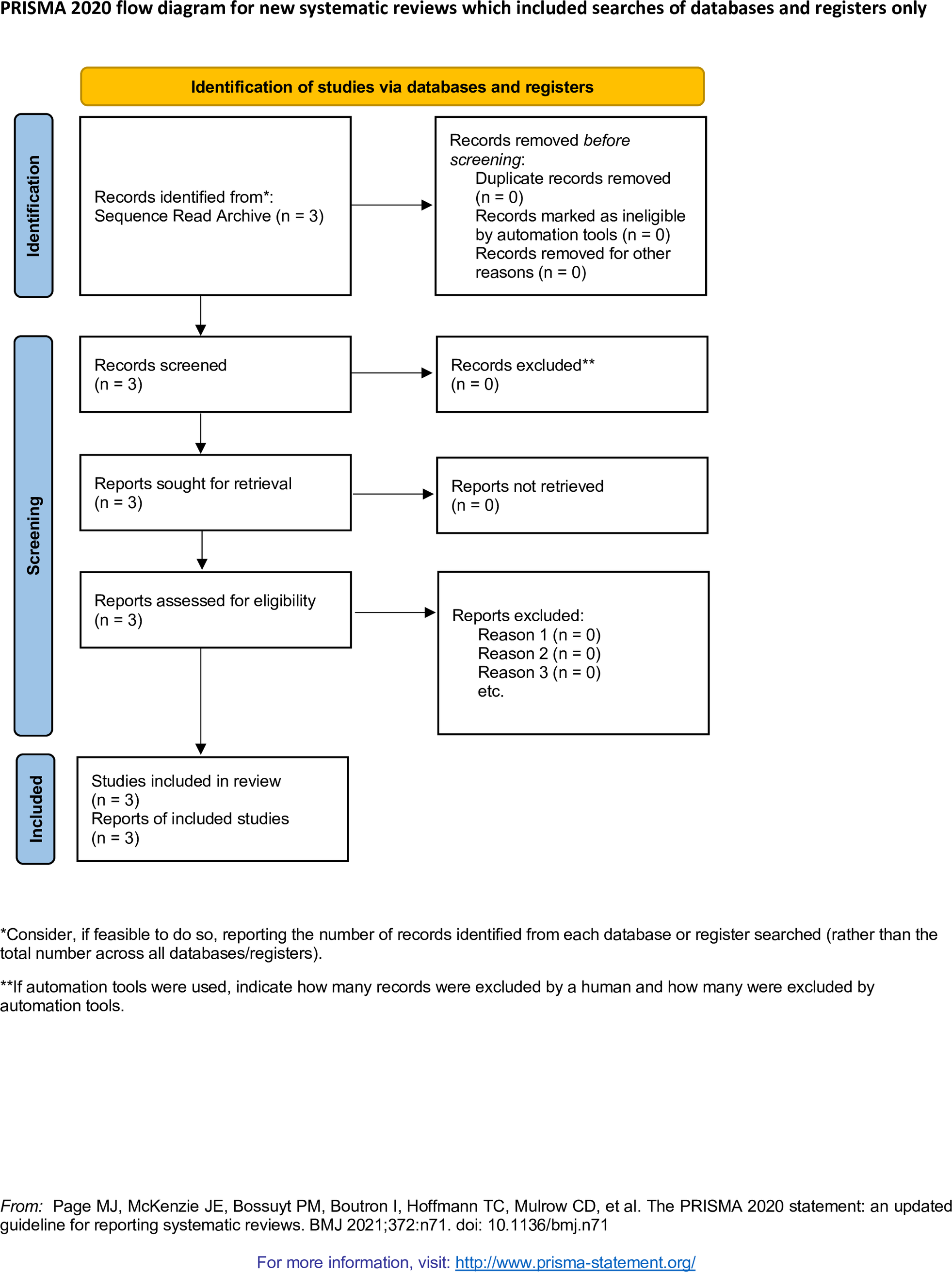

Supplement: S1 Fig — (TIF) [file pone.0295542.s001.tif]
